# Randomised-controlled feasibility trial on abdominal wall closure techniques in patients undergoing relaparotomy (ReLap study; DRKS00013001)

**DOI:** 10.1007/s00423-020-01903-1

**Published:** 2020-06-06

**Authors:** Pascal Probst, Dinh Thien-An Tran, Felix J. Hüttner, Julian C. Harnoss, Patrick Heger, Alina S. Ritter, Colette Doerr-Harim, André L. Mihaljevic, Phillip Knebel, Martin Schneider, Markus W. Büchler, Markus K. Diener

**Affiliations:** 1 Department of General, Visceral and Transplantation Surgery, University of Heidelberg, Im Neuenheimer Feld 110, 69120 Heidelberg, Germany; 2 The Study Center of the German Surgical Society (SDGC), University of Heidelberg, Im Neuenheimer Feld 110, 69120 Heidelberg, Germany

**Keywords:** Abdominal surgery, Abdominal wall, Fascial closure, Relaparotomy

## Abstract

**Background:**

Patients undergoing relaparotomy are generally underrepresented in clinical trials, despite how common the procedure is in clinical practice. Specifically, techniques for re-do abdominal wall closure have never been evaluated in a randomised-controlled trial. The aim of this trial was to identify the optimal abdominal wall closure technique in patients undergoing relaparotomy.

**Methods:**

In this monocentric, randomised feasibility trial, patients scheduled for elective relaparotomy were randomised to abdominal wall closure with either the small stitches technique, using Monomax® 2-0, or the large stitches technique, using PDS II® 1 loop. Patients’ postoperative courses were followed for 1 year after the index operation. Effectiveness and safety outcomes were compared at a level of significance of 5% between the two techniques.

**Results:**

A total of 100 out of 131 patients (76.3%) were evenly randomised to the small stitches and large stitches groups. The time for abdominal wall closure did not differ between the two techniques (small stitches 27.5 ± 9.5 min versus large stitches 25.3 ± 12.4 min; *p* = 0.334). The overall comprehensive complication index was 14.4 ± 15.5 in the small stitches group and 19.9 ± 23.4 in the large stitches group (*p* = 0.168). Specifically, rates of surgical site infection (small stitches 30.0% versus large stitches 36.0%; *p* = 0.524) and burst abdomen (small stitches 4.0% versus large stitches 0.0%; *p* = 0.495) did not differ. After 1 year, incisional hernia rate was 7.5% in the small stitches group and 10.0% in the large stitches group (*p* > 0.999).

**Discussion:**

Both abdominal wall closure techniques investigated in this trial were feasible in relaparotomy patients. This exploratory trial revealed no noticeable difference in the effectiveness or safety of the small stitches technique with Monomax® 2-0 versus the large stitches technique with PDS II® 1 loop. Therefore, surgeons should stay with their preferred suture technique in relaparotomy patients.

**Trial registration:**

Deutsches Register Klinischer Studien (www.germanctr.de): DRKS00013001

**Electronic supplementary material:**

The online version of this article (10.1007/s00423-020-01903-1) contains supplementary material, which is available to authorized users.

## Introduction

Despite advances in minimally invasive surgery, laparotomy remains a mainstay strategy for abdominal access. Relaparotomy is thus frequently necessary, both for malignant and benign recurrent diseases. About 10% of laparotomies are therefore relaparotomies, which pose some unique characteristics [1]. Current evidence suggests that relaparotomy poses a twofold risk of incisional hernia, resulting in higher costs and a reduced quality of life [2–4].

Many trials have sought to identify the ideal method of abdominal wall closure. In 2010, the INLINE meta-analysis concluded that a slowly absorbable continuous suture is preferable [5]. Occurrence of incisional hernia decreased further; after a new suture technique, the small stitches technique was developed [6–8]. However, none of these trials specifically addressed the population of relaparotomy patients, for which the large stitches technique is still frequently applied in clinical practice.

The aim of this trial was to fill this gap by gaining evidence on the feasibility of abdominal wall closure after relaparotomy, comparing the small stitches technique with Monomax® 2-0 with the large stitches technique with PDS II® 1 loop, and to collect data on surgical effectiveness and safety for future confirmatory trials.

## Methods

### Trial design

The ReLap study was planned as a monocentric, prospective, mixed-methods (health care research, translational research, and randomised-controlled trial) exploratory study on patients undergoing relaparotomy. The protocol comprises three steps, the last of which, the randomised-controlled trial, is represented by this manuscript. The trial was conducted at the Clinical Trial Center of the Department of General, Visceral, and Transplantation Surgery at the University of Heidelberg.

The ReLap study was conducted in accordance with the current version of the Declaration of Helsinki [9] and the professional code for physicians in Germany (§15 BOÄ). The study protocol was reviewed and approved by the ethics committee of the medical faculty of the University of Heidelberg (S-442/2017). The study was registered with the German Clinical Trials Register (www.germanctr.de: DRKS00013001) before the first patient was enrolled. The protocol was published in a peer-reviewed open access journal [10]. The trial is reported according to the CONSORT guidelines [11].

### Participants

All patients undergoing a laparotomy were assessed for eligibility. Eligibility criteria were chosen to achieve a broad sample representative of high-volume surgical centres. Patients 18 years or older undergoing any kind of relaparotomy were included, with the following exceptions: Those undergoing relaparotomy for incisional hernia or laparostomy, those having an emergency operation or an operation of the retroperitoneum without transperitoneal access, and incompliant patients were excluded. Before inclusion in the ReLap study, patients were informed about the study orally and gave written informed consent prior to surgery.

### Interventions

The small stitches technique was compared with the large stitches technique. Both techniques have been used as standard forms of closure for primary and relaparotomies at the investigating institution.

#### Abdominal closure with the small stitches technique using Monomax® 2-0

Monomax® 2-0 is an extra slowly absorbing elastic monofilament suture with a thread size of United States Pharmacopeia (USP) 2-0. The first stitch was anchored cranially and caudally of the incision with a knot. The distance from the edge of the fascia was 5 mm and the distance between the two stitches was 2 and 5 mm. Overall, two threads were used; one from the cranial end and the other from the caudal end. Both threads were separately knotted in the middle.

#### Abdominal closure with the large stitches technique using PDS II® 1-loop

PDS II® 1-loop is a slowly absorbing monofilament suture, with a thread size of USP 1, and is formed as a loop. The first stitch was anchored cranially and caudally to the incision. The needle was pulled through the loop so a knot was not necessary. The distance to the edge of the fascia was a maximum of 10 mm and the distance between the two stitches was 15 and 20 mm. Overall, two threads were used; one from the cranial end and the other from the caudal end. Both threads were separately knotted in the middle.

Neither subcutaneous sutures nor subcutaneous drainages were placed in either group. The skin was closed with skin staples. All patients were treated within the standardised fast track concept, which includes physiotherapy-assisted early mobilisation and early transition to a normal diet.

### Outcomes

The postoperative course was followed prospectively with study visits on postoperative days 3 to 7, 10 to 14, and 30. However, if the patient remained in the hospital longer than average or was re-admitted, the course was followed until hospital discharge.

Due to the exploratory nature of this study, there was no primary endpoint. The feasibility of both techniques was assessed based on the rate of included patients versus randomised patients, as determined by the operating surgeon’s clinical judgement. Existing adhesions were evaluated intraoperatively, according to the peritoneal adhesion index, which divides the abdomen into 10 areas to be rated with a number from 0 (no adhesions) to 3 (strong adhesions) [12]. These ratings are summed to produce an index between 0 and 30. Postoperative morbidity and mortality according to the Clavien-Dindo classification were deemed outcomes of interest [13]. The comprehensive complication index [14] was also calculated. Complications that were specifically assessed included burst abdomen, superficial, deep, and organ/ space surgical site infection (SSI) according to the CDC criteria [15], and postoperative haemorrhage. Further, time to first bowel movement, length of hospital stay, and length of stay on the intensive care unit were evaluated.

Patients received a follow-up phone call after 1 year and, if necessary, the patient’s general practitioner was also contacted by telephone. Patients and their general practitioners were asked for the clinical occurrence of an incisional hernia and no radiological proof was demanded. Presence of an incisional hernia, as well as whether or not the hernia required operative treatment, was evaluated after 1 year. The quality of life (EuroQol five-dimensional questionnaire (EQ-5D) [16]) was assessed preoperatively, at day of discharge and after 1 year.

### Sample size

Since no primary endpoint was defined, no sample size was calculated. A total of 100 patients was considered suitable to obtain enough data on feasibility and comparative effectiveness for forming hypotheses for future trials.

### Methods for minimising bias

The randomisation sequence was computer-generated, with a mixture of variable block sizes of 4, 6, 8, and 10. Consecutively, numbered and sealed opaque envelopes containing a card marked “Monomax®” or “PDS II®” were used for allocation. Directly before abdominal wall closure, the operating surgeon evaluated the feasibility of stitching the fascia with both techniques. Reasons for infeasibility were recorded; however, if both techniques were considered feasible, the next envelope in numerical order was opened.

The five study contributors [17] were blinded as follows: Patients were blinded to the suture material. While the operating surgeon could not be blinded to the suture technique, the surgeon played no role in the assessment of outcomes. The data collectors and outcome assessors were aware of the suture material for short-term and long-term outcomes. Statisticians were not blinded to the group allocation; however, the analysis was performed after the closure of the database and according to the published protocol.

### Statistical methods

Data were presented either as mean with standard deviation or as rate. A descriptive *p* value was determined by chi-square test for binary data or Student’s *t* test for continuous data. For burst abdomen and incisional hernia, a relative risk with a 95% confidence interval and number needed to harm (NNH) was calculated to determine the possible futility of future trials. Statistical analysis was performed with R [18].

## Results

**Fig. 1:**
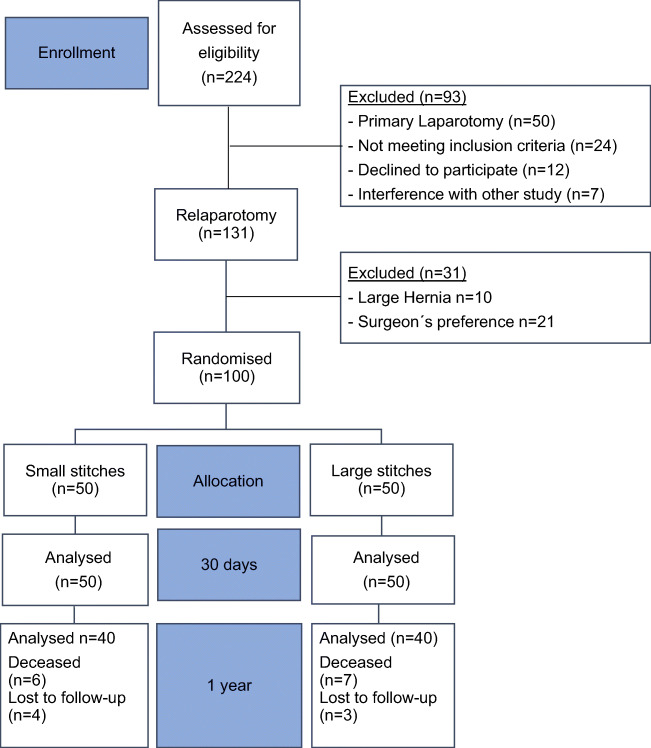
Study flow chart

Recruitment began on September 19, 2017, and the last patient was enrolled on April 4, 2018. The feasibility of both techniques was a given, as 100 of 131 patients (76.3%) could be randomised, indicating a firm conviction of the performing surgeons that both techniques are applicable. The patients not randomised were excluded for the following reasons: 10 patients had an incisional hernia needing a mesh closure, and in the case of 21 patients, the operating surgeon preferred the closure with another technique. Consequently, these 31 patients were closed with large stitches technique or interrupted sutures with or without an additional mesh. No patients were lost to short-term follow-up. However, only 40 of 50 patients (80%) in the small stitches group and 40 of 50 patients (80%) in the large stitches group were available for 1-year postoperative visit. The CONSORT flow diagram is shown in Fig. 1.

### Baseline data

Despite randomisation, two baseline characteristics differed between the small stitches and the large stitches groups. There were more females and more pancreatic resections in the large stitches group. Baseline data are shown in Table 1.

**Table 1:** Baseline characteristics

*N* (%) or mean (SD)	Small stitches (*n* = 50)	Large stitches (*n* = 50)
Sex		
Female	16 (32%)	27 (54%)
Male	34 (68%)	23 (46%)
Age (years)	60.2 (13.1)	62 (10.9)
BMI, kg/m2	24.3 (3.9)	23.9 (3.9)
Charlsons comorbidity index	2.6 (1.7)	2.5 (1.8)
ASA ≥ 3	27 (54.0%)	19 (38.0%)
Days since last op	819.6 (1175.5)	1220.4 (1925.3)
Malignancy as indication	40 (80.0%)	42 (84.0%)
Median laparotomy	49 (98%)	44 (88%)
Resected organ systems*		
Liver	11 (22.0%)	13 (26.0%)
Pancreas	4 (8.0%)	13 (26.0%)
Stomach	5 (10.0%)	9 (18.0%)
Duodenum	2 (4.0%)	8 (16.0%)
Small intestine	20 (40.0%)	25 (50.0%)
Appendix	0 (0.0%)	25 (50.0%)
Colon	13 (26.0%)	10 (20.0%)
Gall bladder	3 (6.0%)	8 (16.0%)
Spleen	0 (0.0%)	1 (2.0%)
Kidney	3 (6.0%)	0 (0.0%)
Gynecologic	0 (0.0%)	3 (6.0%)
Central vessels	3 (6.0%)	3 (6.0%)
Peritoneum	8 (16.0%)	7 (14.0%)
Other	20 (40.0%)	16 (32.0%)

*ASA*, American Society of Anesthesiologists; *BMI*, body mass index

*More than one organ was possible per operation

### Operative data

All patients had an elective relaparotomy. Regarding operative data, there were no differences between the two groups (Table 2). Neither the length of incision (small stitches 26.8 ± 3.9 cm versus large stitches 26.3 ± 4.7 cm; *p* = 0.585) nor the length of the fascial incision (small stitches 27.1 ± 3.7 cm versus large stitches 27.0 ± 4.6 cm; *p* = 0.923) differed. Some patients in both groups had small incisional hernias that were found incidentally, during laparotomy (small stitches 4.0% versus large stitches 8.0%; *p* = 674). Adhesions are common in relaparotomy patients. The severity of adhesions as assessed according to the peritoneal adhesion index was 8.8 ± 5.9 in the small stitches group and 11.4 ± 9.0 in the large stitches group (*p* = 0.095). The time for abdominal wall closure did not differ between the techniques (small stitches 27.5 ± 9.5 min versus large stitches 25.3 ± 12.4 min; *p* = 0.334). Also, the total operative time did not differ (small stitches 201.8 ± 103.1 min versus large stitches 226.1 ± 116.9 min; *p* = 0.272). In both groups, intraabdominal drains were commonly used (small stitches 58.0% versus large stitches 68.0%; *p* = 0.407). The experience level of the operating surgeon performing the fascial closure did not differ, either (small stitches 13.6 ± 7.9 years versus large stitches 13.2 ± 7.3 years; *p* = 0.803).

**Table 2:** Operative data

*N* (%) or mean (SD)	Small stitches (*n* = 50)	Large stitches (*n* = 50)	*p* value*
Skin incision (cm)	26.8 (3.9)	26.3 (4.7)	0.585
Fascia incision (cm)	27.1 (3.7)	27.0 (4.6)	0.923
Hernia	2 (4.0%)	4 (8.0%)	0.674
Peritoneal adhesion index	8.8 (5.9)	11.4 (9.0)	0.095
Operative time (min)	201.8 (103.1)	226.1 (116.9)	0.272
Abdominal wall closure (min)	27.5 (9.2)	25.3 (12.4)	0.334
Intraabdominal drainage	29 (58.0%)	34 (68.0%)	0.407
Surgeon’s experience (years)	13.6 (7.9)	13.2 (7.3)	0.803

*Categorical variables, chi-square test; continuous variables, Student’s *t* test

### Postoperative complications

The overall comprehensive complication index did not differ significantly between the groups (14.4 ± 15.5 in the small stitches group and 19.9 ± 23.4 in the large stitches group; *p* = 0.168). None of the Clavien-Dindo classifications differed (Table 3). Surgical site infection showed no difference between the groups (small stitches 30.0% versus large stitches 36.0%; *p* = 0.524), irrespective of whether the SSI was superficial, deep, or at organ space (Table 3).

**Table 3:** Postoperative complications

*N* (%) or mean (SD)	Small stitches (*n* = 50)	Large stitches (*n* = 50)	*p* value*
According to Clavien-Dindo classification			
I	19 (0.38 pp)	26 (0.52 pp) 0.159	
II	20 (0.40 pp)	29 (0.58 pp) 0.072	
IIIa	9 (0.18 pp)	4 (0.08 pp) 0.234	
IIIb	5 (0.10 pp)	10 (0.02 pp) 0.161	
IVa	0 (0.00 pp)	0 (0.00 pp) > 0.999	
IVb	0 (0.00 pp)	0 (0.00 pp) > 0.999	
V (mortality)	0 (0%)	1 (2%) > 0.999	
CCI	14.4 (15.5)	19.9 (23.4) 0.168	
Surgical site infection	15 (30.0%)	18 (36.0%) 0.524	
Superficial	6 (12.0%)	11 (22.0%) 0.183	
Deep	1 (2.0%)	1 (2.0%) > 0.999	
Organ/space	8 (16.0%)	6 (12.0%) 0.564	
Burst abdomen	2 (4.0%)	0 (0.0%) 0.495	
Incisional hernia at 1 year	3 of 40 patients (7.5%)	4 of 40 patients (10.0%) > 0.999	
Operated for hernia	3 of 40 patients (7.5%)	0 of 40 patients (0%) 0.266	

*CCI*, comprehensive complication index; *pp*, per patient

*Categorical variables, chi-square test; continuous variables, Student’s *t* test

In the small stitches group, 2 of 50 patients (4%) had a burst abdomen compared with 0 of the 50 patients in the large stitches group (*p* = 0.495). The relative risk of developing a burst abdomen with small stitches compared with large stitches was at least 4, with a 95% confidence interval of 0.463 to 34.545. The NNH for the small stitches technique was 25 patients.

One year after the index operation, 3 of 44 patients (6.8%) in the small stitches group and 4 of 43 patients in the large stitches group (9.3%) had an incisional hernia. The relative risk of developing an incisional hernia with small stitches compared with large stitches was 0.75, with a 95% confidence interval of 0.179 to 3.138. The NNH with the large stitches technique was 40 patients. All patients in the small stitches group were operated on for the incisional hernia, whereas none in the large stitches group were, i.e. the relative risk of developing an incisional hernia requiring surgery with small stitches compared with large stitches is at least 3, with a 95% confidence interval of 0.326 to 27.631. The NNH for the small stitches technique was 14 patients.

### Further outcomes

First bowel movement did not differ between the small stitches group (2.6 ± 1.4 days) and the large stitches group (1.6 ± 1.7 days; *p* = 0.798). The length of hospital stay (small stitches 12.2 ± 9.6 days versus large stitches12.5 ± 9.4; *p* = 0.842) as well as the length of stay on the intensive care unit (small stitches 0.8 ± 2.5 days versus large stitches 2.3 ± 7.7; *p* = 0.194) did not differ, either.

### Quality of life

None of the five dimensions of the EQ-5D as well as the scale between 0 and 100 for overall quality of life differed between the two groups at the preoperative visit or at the time of discharge. One year after the index operation, the overall quality of life was better in the large stitches group compared with the small stitches group (68.1 versus 57.1; *p* = 0.025). Overall, the patients in the large stitches group were more independent in their daily living when compared with the small stitches group (92.5% versus 72.5%; *p* = 0.039). Electronic supplementary material 1 gives a detailed overview of quality of life.

## Discussion

The optimal technique for abdominal wall closure of patients undergoing relaparotomy has never been specifically evaluated. Therefore, this randomised-controlled exploratory trial compared 50 patients who received abdominal wall closure with the small stitches technique with Monomax® 2-0 with 50 patients who received abdominal wall closure with the large stitches technique with PDS II® 1 loop.

The final number of randomised patients was important for concluding the feasibility of both techniques in daily practice. Prior to this trial, multiple surgeons in our department had major concerns about the feasibility of closing the abdominal wall with Monomax® thread during relaparotomy. However, this trial showed that in three out of four relaparotomy cases, the operating surgeon deemed that abdominal wall closure would be feasible with either technique. The remaining patients’ incisions were primarily closed with the large stitches technique, as well as with interrupted sutures with or without additional mesh. Therefore, the concern that the small stitches technique with Monomax® was not feasible was unsubstantiated.

The time needed for abdominal wall closure was neither statistically different nor was the difference clinically meaningful between the two techniques. The BMI of included patients was around 24 kg/m^2^ which is comparable with earlier trials [6, 8]. Maybe, the BMI of the normal population would be higher, but a lower BMI could be expected in the relaparotomy population due to malignant recurrent diseases in many cases. There were no differences between the groups regarding 30-day postoperative complications or incisional hernias after 1 year. The overall SSI rate in this trial (30–36%) was higher than those of older trials investigating patients undergoing relaparotomy (6.5–12%) [2]. This variation might be due to differences in the definition of SSI. The presented trial used the CDC criteria and intraabdominal infections, therefore, were considered as SSI. The rate of superficial and deep SSI was comparable with the existing literature [19–22].

Several trials and studies identified relaparotomy as a risk factor for the development of incisional hernia [2, 3, 23, 24]. For example, Lamont et al. described a rate of incisional hernia following relaparotomy of 12% [2]. For primary laparotomy with a closure using the small or large stitches technique, the rate of incisional hernia after 1 year is reported to be 8.5 to 15.7% [5]. In-house data from a trial published 10 years ago have shown an incisional hernia rate of 16% after 1 year with the large stitches technique for primary laparotomy [25]. A multi-centre trial from Germany showed an incisional hernia rate of 8.4% with PDS II® 1-loop and 12.5% with MonoPlus 2-0 in primary laparotomies after 1 year [26]. In the present trial, the incisional hernia rate was between 7.5 and 10%. Therefore, comparing the findings of this trial to literature, the general hypothesis that relaparotomies have higher rates of incisional hernia than primary laparotomies cannot be confirmed.

The evaluation of the quality of life questionnaires showed that the patients in the large stitches group were more independent in their daily living than those in the small stitches group. This finding is unlikely to be related to suture technique. It is more likely that it occurred by chance, due to the comparison of 18 items on the QoL assessment. A second, rather unlikely explanation could be that a lower level of independence was associated with the more clinically relevant incisional hernias in the small stitches group. However, the fact that all patients with an incisional hernia in the small stitches group were operated is very likely caused by chance due to small numbers.

A central question of this exploratory trial was whether or not the data gathered would justify further confirmatory trials. The relative risk for burst abdomen and for a clinically relevant incisional hernia was higher in the small stitches group. The 95% confidence interval, as well as the NNH, is in an area of clinically relevant effect estimates. For a confirmatory trial showing the superiority of the large stitches technique in respect of incisional hernia in relaparotomy patients, about 400 patients would be needed. However, such a study should not be undertaken now due to the on-going HULC trial, which investigates the purported benefits of prophylactic onlay mesh for laparotomy and includes also relaparotomy patients [27]. These results should be obtained before a confirmatory trial for relaparotomy patients alone is undertaken.

This RCT has several limitations. As this was an exploratory and monocentric trial, the absence of significant differences should not be confused with the potential for greater differences in a confirmatory setting, as discussed above. Further baseline differences in the PDS II® group, that of more females and more pancreatic resections, may have confounded results. Furthermore, all surgeons were asked to perform a minimum suture length to wound length ratio of 4:1, however, there was no monitoring of performance. Finally, it is known that incisional hernia rates increase by about 60% from assessment at 1 year to assessment after 3 years [28], so these results may not adequately describe the final incisional hernia rate. Moreover, this trial assessed incisional hernias only by a phone call with the patient and his general practitioner. Therefore, the rate of incisional hernias is limited to clinically obvious hernias. This pragmatic approach was chosen because of the feasibility nature of the presented trial but would not be adequate in a confirmatory setting.

To summarise, both the small and large stitches techniques are feasible in relaparotomy patients without producing relevant differences in operative time or morbidity. Based on the presented data, there is no need to advocate a general change in abdominal wall closure, and surgeons should stay with their preferred suture technique. However, if surgeons prefer the small stitches technique during primary laparotomy, there is no need to switch to another technique or thread for relaparotomy patients. Due to the on-going interventions in the ideal type of abdominal wall closure, a confirmatory trial comparing the small stitches technique using Monomax® 2-0 with the large stitches technique using PDS II® 1 loop is not recommended at the moment.

## Electronic supplementary material

ESM 1 (PDF 60 kb)
