# Supplementary material for: Randomised-controlled feasibility trial on abdominal wall closure techniques in patients undergoing relaparotomy (ReLap study; DRKS00013001)
Source: Langenbecks Arch Surg. 2020 Jun 6;405(4):427–34. doi: 10.1007/s00423-020-01903-1 (PMC7359135; doi:10.1007/s00423-020-01903-1)
Supplement: Supplementary file 1 — (PDF 60 kb) [file 423_2020_1903_MOESM1_ESM.pdf]

Quality of life<br>EuroQol five-dimensional questionnaire	Preoperative			Hospital discharge			One Year		
**N (%) or mean (SD)**	**Small stitches (n=50)**	**Large stitches (n=50)**	**p-value***	**Small stitches (n=50)**	**Large stitches (n=50)**	**p-value***	**Small stitches (n=40)**	**Large stitches (n=40)**	**p-value***
**Mobility**									
no problems	44 (88.0%)	47 (94.0%)	0.485	42 (84.0%)	37 (74.0%)	0.248	31 (77.5%)	33 (82.5%)	0.957
some problems	6 (12.0%)	3 (6.0%)		8 (16.0%)	11 (22.0%)		8 (20.0%)	7 (17.5%)	
many problems	0 (0.0%)	0 (0.0%)		0 (0.0%)	2 (4.0%)		1 (2.5%)	0 (0.0%)	
**Independence**									
no problems	46 (92.0%)	47 (94.0%)	> 0.999	44 (88.0%)	41 (82.0%)	0.630	29 (72.5%)	37 (92.5%)	0.039
some problems	4 (8.0%)	3 (6.0%)		4 (8.0%)	7 (14.0%)		11 (27.5%)	3 (7.5%)	
many problems	0 (0.0%)	0 (0.0%)		2 (4.0%)	2 (4.0%)		0 (0.0%)	0 (0.0%)	
**Daily Tasks**									
no problems	34 (68.0%)	37 (64.0%)	0.428	25 (50.0%)	24 (48.0%)	0.936	19 (47.5%)	20 (50.0%)	>0.999
some problems	16 (32.0%)	12 (24.0%)		21 (42.0%)	21 (42.0%)		17 (42.5%)	18 (45.0%)	
many problems	0 (0.0%)	1 (2.0%)		4 (8.0%)	5 (10.0%)		4 (10.0%)	2 (5.0%)	
**Pain**									
no problems	32 (64.0%)	27 (44.0%)	0.475	27 (34.0%)	23 (26.0%)	0.301	24 (60.0%)	19 (47.5%)	0.417
some problems	16 (32.0%)	20 (40.0%)		23 (26.0%)	25 (50.0%)		13 (32.5%)	17 (42.5%)	
many problems	2 (4.0%)	3 (6.0%)		0 (0.0%)	2 (4.0%)		3 (7.5%)	4 (10.0%)	
**Fear**									
no problems	25 (50.0%)	28 (56.0%)	0.858	31 (62.0%)	26 (32.0%)	0.311	19 (47.5%)	22 (55.0%)	>0.999
some problems	20 (40.0%)	19 (38.0%)		14 (28.0%)	21 (42.0%)		16 (40.0%)	17 (42.5%)	
many problems	5 (10.0%)	3 (6.0%)		5 (10.0%)	3 (6.0%)		5 (12.5%)	1 (2.5%)	
**Overall**	60.6 (20.3)	66.1 (20.7)	0.184	59.0 (21.5)	58.2 (25.9)	0.715	57.1 (23.2)	68.1 (19.8)	0.025

*Categorical variables: Chi-squared test; continuous variables: student´s t-test
